# Immature symbiotic system between horizontally transmitted green algae and brown hydra

**DOI:** 10.1038/s41598-021-82489-6

**Published:** 2021-02-03

**Authors:** Ryo Miyokawa, Hiroyuki J. Kanaya, Taichi Q. Itoh, Yoshitaka Kobayakawa, Junko Kusumi

**Affiliations:** 1 Graduate School of Integrated Science for Global Society, Kyushu University, 744 Moto-oka, Nishi-ku, Fukuoka, 819-0395 Japan; 2 School of Science, Kyushu University, 744 Moto-oka, Nishi-ku, Fukuoka, 819-0395 Japan; 3 Faculty of Arts and Science, Kyushu University, 744 Moto-oka, Nishi-ku, Fukuoka, 819-0395 Japan; 4 Department of Environmental Changes, Faculty of Social and Cultural Studies, Kyushu University, 744 Moto-oka, Nishi-ku, Fukuoka, 819-0395 Japan

**Keywords:** Evolution, Evolutionary developmental biology, Evolutionary genetics

## Abstract

Some strains of brown hydra (*Hydra vulgaris*) are able to harbor the green algae *Chlorococcum* in their endodermal epithelial cells as symbionts. However, the relationship between brown hydra and chlorococcum is considered to be incipient symbiosis because most artificially introduced symbionts are not stable and because symbiotic *H. vulgaris* strains are rare in the wild. In this study, we compared the gene expression levels of the newly established symbiotic hydra (strain 105G), the native symbiotic strain (J7), and their non-symbiotic polyps to determine what changes would occur at the early stage of the evolution of symbiosis. We found that both the 105G and J7 strains showed comparable expression patterns, exhibiting upregulation of lysosomal enzymes and downregulation of genes related to nematocyte development and function. Meanwhile, genes involved in translation and the respiratory chain were upregulated only in strain 105G. Furthermore, treatment with rapamycin, which inhibits translation activity, induced the degeneration of the symbiotic strains (105G and J7). This effect was severe in strain 105G. Our results suggested that evolving the ability to balance the cellular metabolism between the host and the symbiont is a key requirement for adapting to endosymbiosis with chlorococcum.

## Introduction

Symbiotic algae behave as mutualistic, parasitic, or free-living organisms, depending on host and symbiont genotypes and environmental conditions^1,2^. Organisms with algal symbionts are widely distributed among various taxonomic groups. Hosts can acquire photosynthates from symbionts, and the symbionts are supplied with nitrogen and carbon sources from their hosts. Hosts also provide symbiotic algae with the benefit of host shelters, which protect the symbionts from predators and environmental fluctuations^3,4^. Such algal endosymbiosis is inferred to have evolved from predator–prey or host–parasite interactions, but the evolutionary processes enabling symbiosis have not been elucidated to date^5^.

Many cnidarian species exhibit symbiotic relationships with algae. For instance, the symbiosis of reef-building corals and sea anemones with zooxanthellae has been intensively studied^6^. Additionally, some anemones and hydras are known to have symbiotic relationships with green algae^7,8^. A stable symbiotic relationship between *Hydra viridissima* (known as green hydra) and *Chlorella* is well-known, and molecular clock analysis has indicated that their symbiotic relationship first appeared more than 77 million years ago^9^. Moreover, several strains of brown hydra (*H. vulgaris*) collected in Japan exhibit *Chloroocccum* sp. as an endosymbiont in their endodermal cells^10^ (e.g., strains J7 and J10). Only the *Chloroocccum* sp. is known to be able to establish the symbiotic relationship with brown hydra among algae species in the genus *Chlorococcum*^10^. Interestingly, many strains of *H. vulgaris* can incorporate the symbiotic algae by artificial introduction to the gastric cavity^11,12^, despite the rarity of symbiotic *H. vulgaris* strains in the wild. However, most of the artificially introduced symbionts are not stable in most host strains, and the tolerance to starvation of the host polyp is decreased in the symbiotic strains of *H. vulgaris* compared with the non-symbiotic strains^11,13^. This finding suggests that the symbiosis between *H. vulgaris* and *Chloroocccum* is less stable than that between *H. viridissima* and *Chlorella*. Ishikawa et al.^12^ suggested the "two-step evolution" of endosymbiosis: the common ancestor had previously obtained endosymbiotic potential with *Chlorococcum*, and the native symbiotic strains obtained symbiotic chlorococci recently. The authors interpreted that the non-symbiotic strains with endosymbiotic potential had not been fully adapted to the symbiosis. In this respect, the hydra–chlorococcum interaction is considered to be a suitable system for elucidating the evolution of symbiosis at an early stage. In a previous study^14^, we showed that non-symbiotic hydras can acquire symbiotic chlorococci through the surrounding water by horizontal transmission without artificial introduction. In addition, the symbiotic hydra strain established by horizontal transmission exhibited notable changes, displaying decreased polyp size and elevated growth rate by budding.

In this study, using RNA-seq, we analysed the gene expression of the symbiotic strain acquired by horizontal transmission, as well as that of the original non-symbiotic strain. We investigated the mechanisms of hydra–chlorococcum symbiosis, which is still in progress, based on the gene expression observed in the acquired symbiotic strain. To elucidate the mechanism governing adaptation to symbiosis in the wild, we compared gene expression changes in the symbiotic states between the acquired symbiotic strain and the native symbiotic strain. We also observed the effects of changes in cellular metabolism between the non-symbiotic strain and the symbiotic strains. These results may help to characterize the early steps of symbiosis evolution.

## Results and discussion

### Comprehensive gene expression profiling of symbiotic and non-symbiotic polyps

To analyse the changes in cellular mechanisms that were induced by horizontal transmission in the symbiotic polyps, we compared the gene expression patterns of the symbiotic hydra strain 105G to those of the original non-symbiotic strain 105. We obtained 137 million paired-end sequence reads after quality control was performed. The proportions of mapped reads of strains 105G and 105 were 69.5% and 79.3%, respectively, and GC content of the mapped reads was 35% for both strains. Green algae generally have a higher GC content level (e.g. *Chlorococcum* sp.2 FFG039: 62%)^15^, so the equivalent GC content suggested contaminated reads of the chlorococci were filtered from the mapped reads of the 105G samples. Out of the total of 31,725 contigs in the reference, 26,696 contigs and 27,001 contigs were expressed in 105G and 105, respectively, and 27,406 contigs were expressed in either strain. In strain 105G, 2,742 contigs were upregulated, and 2,971 contigs were downregulated, compared to strain 105. In the expressed contigs, 11,467 contigs could be annotated with descriptions of *H. vulgaris* genes in RefSeqGene, excluding uncharacterized genes. In addition, we performed similarity searches using BLASTX to assign functional annotations to the mapped genes. As a result, 6,080 contigs were matched with entries in UniProtKB Swiss-Prot, and 807 contigs were matched with entries for *H. vulgaris* in UniProtKB TrEMBL.

**Figure 1:**
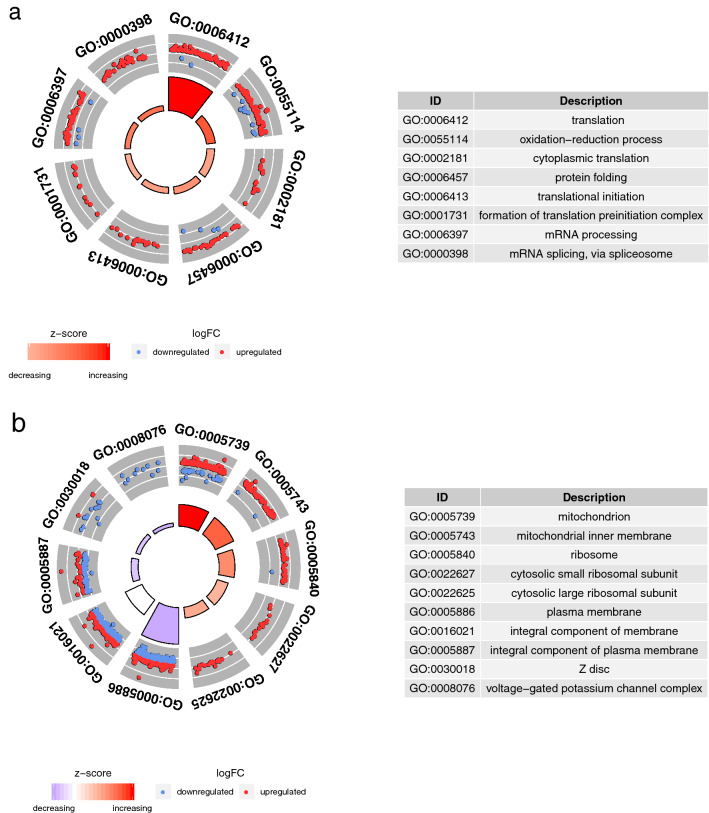
Functionary characterization of differentially expressed genes (DEGs). The outer circle shows scatter plots of the expression levels of DEGs in each enriched GO term. The inner circle represents the z-scores (differences between the number of upregulated DEGs and downregulated DEGs) by colour and the adjusted *P*-values by height. (**a**) Top 8 enriched GO terms in the BP category. (**b**) Top 5 enriched GO terms in each upregulated and downregulated gene in the CC category.

We performed Gene Ontology (GO) enrichment analysis using the DAVID Functional Annotation tool and illustrated enriched Gene Ontology terms as GOCircle plots^16^ (Fig. 1). Among the upregulated genes, 31 GO terms were significantly enriched, while 8 GO terms were enriched among the downregulated genes (Supplementary Tables S1, S2). In the biological process (BP) category, 8 GO terms were significantly enriched in the upregulated genes (Fig. 1a). In particular, translation exhibited the highest z-score (*Z* = 8.20) and the lowest *P*-value (*P* = 1.18e−14) among the enriched categories. In cellular component (CC) categories, 22 GO terms were significantly enriched (16 upregulated and 6 downregulated). We present the details for five of the GO terms in the CC category with higher *P*-values in the upregulated and downregulated genes, respectively, in Fig. 1b. Mitochondria had the highest z-score (*Z* = 10.27) and a lower *P*-value (*P* = 2.24e-10). Differentially expressed genes (DEGs) in the GO term mitochondrion, such as respiratory chain complexes, overlapped with DEGs in the GO term oxidation–reduction process.

In host organisms that have green algae (chlorellae) as endosymbionts, namely, *H. viridissima* and *Paramecium bursaria*, translation is the enriched GO term in downregulated genes in the symbiotic state^13,17^. Ishikawa et al.^13^ interpreted that the respiratory chain process in the cells in *H. viridissima* is inactivated to suppress the generation of harmful reactive oxygen species (ROS). Furthermore, Kodama et al.^17^ demonstrated that protein biosynthesis in *P. bursaria* cells is controlled by substance exchange between the hosts and the symbionts. In these well-established symbioses, it is suggested that cooperative biosynthesis and/or substance exchange between the host cells and the symbiont algae have been established. In our results for 105G/105, however, the genes assigned to the GO terms translation, mitochondrion, and oxidation–reduction process were upregulated. The increase in metabolic activity may be a physiological phenomenon due to increased oxygen and/or nutrient supply by the symbionts, and the cooperative and adaptive responses to the symbionts may not have yet occurred in the newly formed symbiotic strain 105G.

### Activation of transcription factors that regulate head and foot formation in the symbiotic hydra acquired by horizontal transmission

Mortzfeld et al*.*^18^ suggested that the maximum polyp size of *Hydra* is primarily determined by the number of cells, not the size of the cells, in a polyp. These researchers showed that Wnt signalling activates TGF-β signalling, that TGF-β signalling initiates budding and that polyp size growth stops when budding begins^18^. The acquired symbiotic strain, 105G, exhibits a decreased polyp size compared with the original strain, 105^14^. A difference in the size of endodermal epithelial cells was not observed between strains 105G and 105 (Supplementary Table S3). In the strain 105G, several genes in the Wnt pathway were differentially expressed (Supplementary Fig. S1). Downstream of the Wnt pathway, transcription factor 7-like 2 (TCF/LEF) activates transcription by forming a complex that binds to β-catenin^19^. Wnt and TCF/LEF are co-expressed in hydra heads and induce bud initiation and head formation^20,21^. Therefore, an expression change of TCF/LEF can fluctuate the time of bud initiation and the developmental time of a polyp. The serine/threonine-protein kinase NLK inhibits the binding of TCF/LEF to DNA and suppresses transcription activation by TCF/LEF^22^. In the acquired symbiotic strain 105G, TCF/LEF was upregulated, and NLK was downregulated (Supplementary Fig. S1). This result indicates that transcription stimulated by TCF/LEF is activated in the acquired symbiotic hydra. TGF-β signalling causes retinoblastoma-like protein 1 (p107) and transcription factor Dp-1 to regulate the transcription activity of transcription factor E2F. E2F activity is inhibited by p107, and this protein is formed by the dimerization of E2F and Dp-1^23,24^. p107 was downregulated, and Dp-1 was upregulated, in strain 105G (Supplementary Fig. S1). This result indicates that transcription of E2F is also activated in strain 105G. Furthermore, we considered the upregulation of the homeobox genes, which are also transcription factors, as other factors that may alter polyp size. Dlx1 and NK-2 are homeobox genes that are expressed in the foot in *Hydra*. Dlx1 is upregulated during foot formation in ectodermal cells^25,26^. NK-2 is expressed in endodermal epithelial cells prior to foot formation^27^. These homeobox genes are activated by Wnt/β-catenin signalling, as well as TCF/LEF, and regulate targeted genes^28^. Wnt signalling might activate the initiation and development of budding via these transcription factors. Paired mesoderm homeobox protein 2 (OtxB) is another homeobox gene that was observed to be upregulated in strain 105G (Supplementary Fig. S1) and expressed in endodermal cells according to single-cell RNA-seq data from *Hydra*^29^. OtxB is a close homologue to three Otx genes in *Nematostella vectensis*. The Otx genes in *N. vectensis* are expressed in the endoderm of the foot and pharynx and in the ectoderm of the tentacle and are involved in endodermal development and patterning^30^. OtxB might be involved in endodermal development according to the function of homologous genes. The upregulation of the transcription factors TCF/LEF, Dlx1, and NK-2 suggests that the horizontal transmission of chlorococci to hydra activates transcription by TCF/LEF and homeobox genes, and it is possible that the decrease in polyp sizes and the increase in asexual reproduction rates in the acquired symbiotic strain 105G depend on these expression changes. However, it has not been determined how these transcription factors control bud development by Wnt signalling.

### Similarity of gene expression involved in phagocytosis and nematocytes between the acquired symbiotic strain and the native symbiotic strain

Ishikawa et al.^12^ postulated that hydras that did not have “endosymbiotic potential” with *Chlorococcum* evolved into native symbiotic hydras in a step-by-step process by first becoming hydras with endosymbiotic potential. Some *Hydra* strains with endosymbiotic potential can acquire symbionts by artificial or spontaneous transmission^10–12,14^. However, it is not clear what kind of step-by-step changes in the cellular mechanisms are required to obtain the endosymbiotic potential. Therefore, we investigated the difference in gene expression patterns between the acquired symbiotic strain 105G and the native symbiotic strain J7 to help elucidate the mechanism governing the establishment of symbiosis.

**Figure 2:**
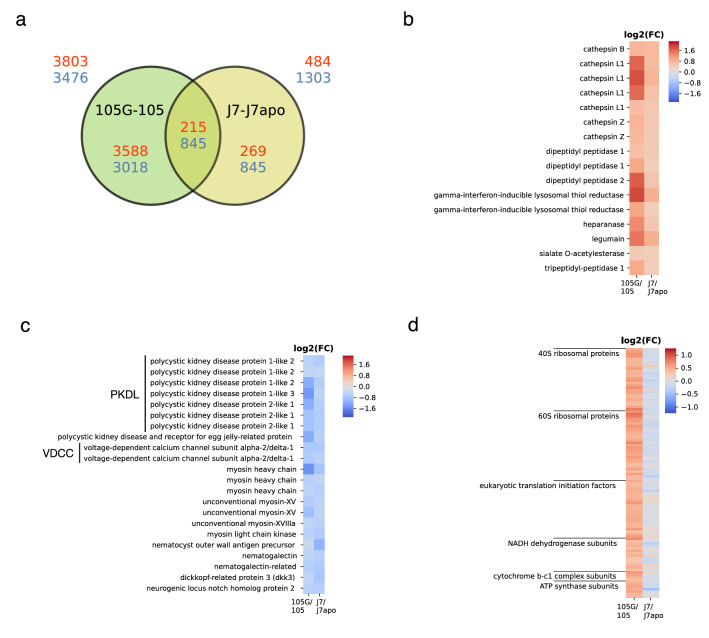
Comparison of the gene expression patterns in the 105G/105 and J7/J7apo pairs. (**a**) Venn diagram showing the number of differentially expressed genes (DEGs) in both strain pairs with the frag-bias-correct option (FDR < 0.05). The numbers of upregulated genes are indicated in red, and the numbers of downregulated genes are indicated in blue. (**b**) Heat map showing log2 fold change of the FPKMs (fragments per kilobase of exon per million mapped reads) of the upregulated genes coding lysosomal enzymes in both strain pairs. (**c**) Heat map showing log2 fold change of the FPKMs of the downregulated genes expressed in nematocytes or playing a role in nematocyte differentiation in both strain pairs. (**d**) Heat map showing log2 fold change of the FPKMs of the upregulated genes in the 105G/105 strain pair, encoding the genes related to translation and respiratory chain complex.

The RNA-seq data of J7 and J7apo strains sequenced by Ishikawa et al.^13^ were compared with those of 105 and 105G strains. J7apo strain is a non-symbiotic strain originated from the J7 strain by artificial elimination of symbionts. The sequence data of each sample was analysed using the Cufflinks pipeline with the frag-bias-correct option to improve the accuracy of expression estimates across RNA-seq libraries^31^. According to the bias correction method, there were 7,279 DEGs between 105G and 105 and 1,787 DEGs between J7 and J7apo (Fig. 2a). Although there was a significant power difference between the cases of 105G/105 and J7/J7apo (105G and 105 strains: 3 replicates each; J7 and J7apo strains: 2 replicates each), the DEGs exhibited a four-fold difference between the 105G/105 and J7/J7apo pairs. We found 673 overlapping DEGs between the 105G/105 and J7/J7apo (Fig. 2a).

In strain J7, one GO term was enriched in the upregulated genes, namely, lysosome (*P* = 2.58e-7), and five GO terms were enriched in the downregulated genes (Supplementary Table S4). The GO term lysosome was also enriched in the upregulated genes in the symbiotic strain J7 in a previous study^13^. We found 16 commonly upregulated genes encoding lysosomal enzymes in the symbiotic strains 105G and J7 (Fig. 2b, Supplementary Table S5). These enzymes encode upregulated genes, except for heparanase and sialate *O*-acetylesterase, which act as proteases. In *H. viridissima*, most of the symbiotic algae are digested in host lysosomes during infection^32^, and the symbiotic algae are digested in the host cells to maintain the number of algal cells^33^. These algal cells can be digested inside enveloped vacuoles by lysosomal enzymes. Similarly, the upregulation of the lysosomal enzymes in the symbiotic *H. vulgaris* could be responsible for the digestion of the symbiotic chlorococci, suggesting that symbiotic *H. vulgaris* may have the potential to employ their lysosomes to digest the symbionts as a resource and/or to maintain a constant cell density of the symbionts in the host cells. We found gene expression changes in strain 105G in the endocytosis and phagocytosis pathways in the KEGG pathway (Supplementary Fig. S2). The neural Wiskott-Aldrich syndrome protein (N-WASP) and the actin-related protein 2/3 complex (Arp2/3) complex play essential roles in polymerization of actin filament in clathrin-mediated endocytosis and phagocytosis^34,35^. Rab7 induces fusion of late endosomes and phagosomes with lysosomes in the presence of damaged symbionts in *Aiptasia*^36^. The upregulation of N-WASP, Arp2/3, and Rab7 may be involved in the uptake of symbionts by phagocytosis and the maintenance of the number of symbionts in host cells.

Among the overlapping DEGs between the 105G/105 and J7/J7apo annotated from RefSeqGene, there were 15 DEGs with log2 fold changes higher than 1 and 2 DEGs with log2 fold changes lower than -1 in both 105G/105 and J7/J7apo (Supplementary Table S6). l-rhamnose-binding lectin CSL3-like showed the highest FC in both strain pairs, and d-galactoside-specific lectin was one of the upregulated genes in both strain pairs. Both lectins belong to the rhamnose-binding lectin (RBL) family according to BLAST searches. RBLs play a role in innate immune recognition to bind l-rhamnose and d-galactose resides in polysaccharides and induce an increase in phagocytic activity^37,38^. In the stony coral *Pocillopora damicornis*, RBL recognizes both pathogenic bacteria and symbiotic algae by binding to polysaccharides on the cell wall surfaces^39^. Galactose is among the major components of a cell wall in *Chlorococcum*, and the cell wall also contains some rhamnose^40^. Furthermore, the symbiotic *Chlorococcum* is agglutinated under the influence of a galactose binding lectin^41^. If RBLs also play a role in the recognition and uptake of the endosymbionts in *H. vulgaris*, the upregulation of the genes in the pathway from phagocytosis to intracellular digestion may reflect host reactions to the presence of the symbionts, extending from uptake to digestion.

The plasma membrane exhibited the lowest *P*-value (*P* = 6.20e−17) among the GO terms in the CC category (Fig. 1b, Supplementary Table S2). Similarly, Ishikawa et al.^13^ reported that the GO term plasma membrane is enriched in downregulated genes in the symbiotic state of the native symbiotic strain J7. Calcium ion binding and integral component of membrane appeared in the enriched GO term in the downregulated genes in both 105G and J7 strains (Supplementary Tables S2 and S4, respectively). Polycystic kidney disease proteins (PKDLs) and voltage-dependent calcium channels (VDCCs) were observed to be commonly downregulated in 105G and J7 strains (Fig. 2c, Supplementary Table S7). PKDLs are non-selective cation channels that have permeability to Ca^2+^^42^, while VDCCs are voltage-gated ion channels with selective permeability to Ca^2+^^43^, and these products are expected to be localized in the plasma membrane according to the Uniprot annotation (UniProtKB, https://www.uniprot.org). PKDLs belong to the polycystin cation channel family and have sequences similar to those of other polycystins. Ishikawa et al.^13^ proposed the hypothesis of Ca^2+^ homeostasis disruption due to the inhibition of genes related to polycystin in endodermal cells with symbionts. However, most DEGs coding VDCCs and related to polycystins, such as PKDLs, are expressed in nematocytes according to single-cell RNA-seq data from *Hydra*^29^. Polycystins and VDCCs were confirmed to be involved in nematocyst discharge^44,45^. The downregulation of these calcium ion channel genes in symbiotic states is probably due to changes in nematocytes, rather than disruption of calcium homeostasis, in endodermal cells containing symbionts. In fact, the stenoteles, which are the largest types of nematocyst, in strain 105G are smaller than the stenoteles in the original non-symbiotic strain 105, and the number of stenoteles per tentacle and the length of tentacles were decreased in strain 105G^14^. In addition, myosins and nematogalectins were downregulated in 105G and J7 strains (Fig. 2c, Supplementary Table S7). These myosins and nematogalectins were mainly expressed in nematocytes and battery cells and nematoblasts, respectively, according to the single-cell RNA-seq data of *Hydra*^29^. These proteins play important roles in nematocyst maturation^46,47^. The downregulation of the genes expressed in nematoblasts and nematocytes in 105G strain is consistent with the above report^14^ of the reduction in stenotele size and count in this strain. This inhibition of nematocyte development and the reduction in stenotele size and count may cause a decrease in prey capture ability in strain 105G. Our previous study revealed that the acquired symbiotic strain 105G has an increased asexual reproduction rate under the high-light condition compared to the non-symbiotic strain 105, while the polyp size of 105G strain was smaller than that of 105 strain^14^. The increase in the asexual reproduction rate in strain 105G might be explained by utilization of the photosynthetic products of symbionts as nutrients in the host. However, the reduction of the polyp size simultaneously occurs in strain 105G, therefore it is still unclear whether the symbiotic chlorococci supply sufficient nutrients to their host.

Ishikawa et al.^13^ proposed that the instability of the symbiosis in strain J7 was derived from the downregulation of gene expression related to cell adhesion molecules. In this study, coadhesin, zonadhesin, protocadherin (Fat cadherin), and protocadherin-like proteins (Fat-like cadherin) were downregulated in the symbiotic strains 105G and J7 (Supplementary Fig. S3). Fat and Fat-like cadherin are transmembrane proteins that have structures that are evolutionarily conserved from cnidarians to mammals, and these products are involved in tissue growth and planar cell polarity in the adherens junctions^48,49^. These cadherin genes were mainly expressed in the battery cells according to the single-cell RNA-seq data of *Hydra*^29^. Battery cells surround such nematocytes as stenoteles and play a role in docking sites of nematocyst vesicles to anchor the nematocytes to the basement membrane on tentacles^50^. Nematocytes are bound to battery cells by adherens junctions^51^, where Fat and Fat-like cadherin are located. The downregulation of Fat and Fat-like cadherin might reflect the reduction of adherens junctions as a reduction in stenotele size and count due to symbiosis.

### Differences in the regulation of cellular metabolism between the acquired symbiotic strain 105G, the native symbiotic strain J7, and stable symbiotic organisms

There were 73 upregulated genes with the GO term translation in strain 105G, while no genes with the GO term translation were upregulated in strain J7. We compared the fold changes of genes related to translation and respiratory chain complexes in the symbiotic state between the 105G and J7 strains. Figure 2d shows the genes upregulated only in strain 105G. All the upregulated genes with the GO term translation in 105G strain did not show significant expression changes in strain J7 (cf. Supplementary Table S8). Translation and mitochondrial activity reciprocally interact and control energy balance in cells through ATP production and consumption^52^. The difference in the patterns of expression changes of translation and respiratory chain complexes between the 105G and J7 strains may reflect different levels of cellular energy production and the changes in substance exchanges between the hosts and the symbionts. Nutritional signals activate TOR complex 1 (TORC1) via the TOR pathway, which is a pathway that is conserved from yeast to mammals^53^. In strain 105G, some genes in the TOR pathway, such as ras-related GTP-binding proteins (rag GTPases: ragA/B, ragC/D) and LAMTOR2, were upregulated (Supplementary Fig. S4). LAMTOR2 is a part of the regulator complex and composes a binding platform to the rag GTPase^54^, and Rag GTPases bind to the regulator complex and activate TORC1 in response to the amino acid signal from lysosomes^55,56^. TORC1 activation induces translation activity, including ribosome biogenesis, and inhibits autophagy^57^. In the symbiotic sea anemone *Aiptasia*, amino acids synthesized by symbiotic algae from photosynthetic products are translocated to host cells^58^, and Voss et al*.*^59^ reported that nutrients from symbionts activate TORC1 signalling and that signalling prompts translation and metabolism in the host cell. However, the authors reported that genes related to lysosomes and translation are downregulated in symbiotic *Aiptasia*, similar to the symbiotic organisms mentioned above, *H. viridissima*^13^ and *P. bursaria*^17^. This finding implies that there are other systems to inhibit translation and cellular metabolism in stable symbiotic organisms, while *H. vulgaris* may not have these systems. Alternatively, the symbiotic *H. vulgaris* might have a reason not to inhibit translation activity to maintain the symbiosis. *Chlorococcum* can use peptone, which is an enzyme digest of animal protein containing amino acids, as a nitrogen source, as well as other general nitrogen sources^60^. If the symbiotic chlorococci take up amino acids from the host cells, ignoring the metabolic needs of the host, the host will have to pay the cost of synthesizing additional amino acids. Several enzymes synthesizing amino acids and involved in the TCA cycle, which generates precursors of amino acids, were upregulated in strain 105G (Supplementary Fig. S5). Thus, the effect on cellular metabolism caused by endosymbiosis would differ among the acquired symbiotic strain 105G, the native symbiotic strains J7, and other stable symbiotic organisms.

**Figure 3:**
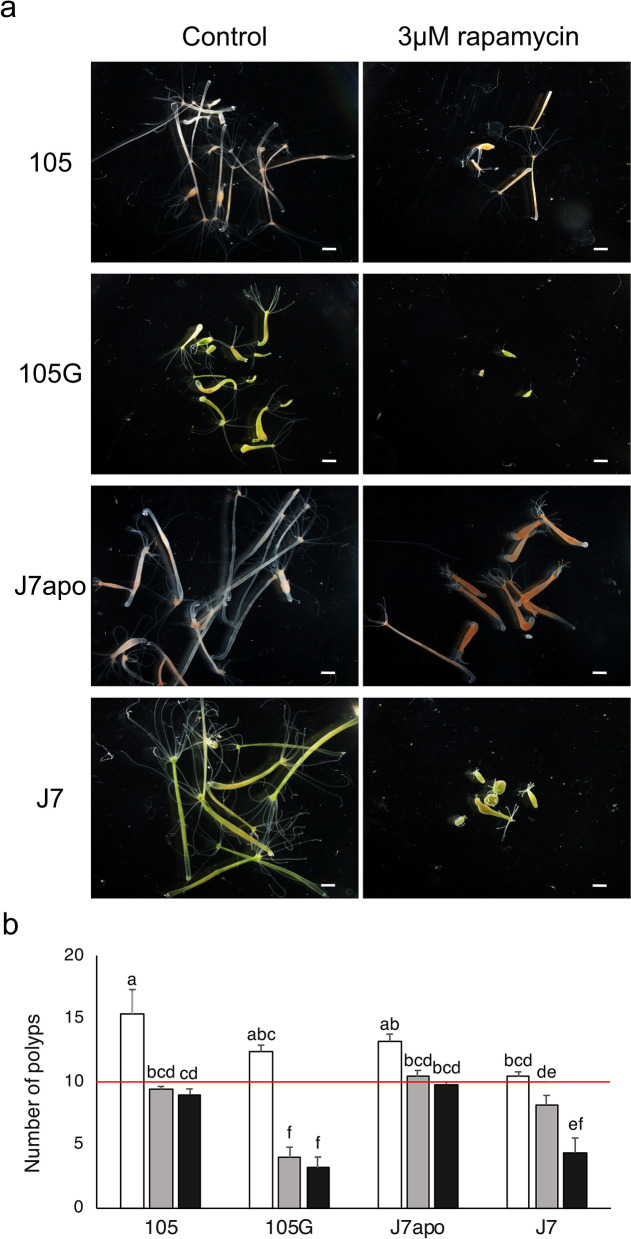
Effect of translation inhibition with rapamycin treatment. (**a**) The polyp condition 2 weeks after the start of rapamycin treatment. The symbiotic polyps (strain 105G, J7) shrank in 3 μM rapamycin. Scale bars: 1 mm. (**b**) The number of polyps two weeks after the start of rapamycin treatment. The error bars show the standard error in each condition. The red line represents the number of polyps (n = 10) at the start of the treatment. The significant differences were calculated by Tukey’s test among each condition (P < 0.05). The same letters between the bars represent no significant difference.

If the regulations of the genes related to translation and metabolism are vital to maintaining the symbiotic system with chlorococci, inhibition of these mechanisms is expected to cause different effects on the symbiotic hydras and the non-symbiotic hydras. We then conducted treatment with rapamycin to inhibit TORC1 activity^61^, regulating translation and metabolism in host cells^59^. Previous studies in *Hydra* reported that short-term exposure to rapamycin (12 h, 10 μM) reduces polyp size^62^ and that long-term exposure to rapamycin (> 30 days, 0.8 μM) delays ageing and improves epithelial proliferation in a strain that senesces upon gametogenesis^63^. We exposed 10 non-symbiotic and symbiotic polyps of the 105, 105G, J7apo, and J7 strains to rapamycin for two weeks with five replicates. Most of the polyps exposed to rapamycin shrank and shortened their tentacles, and some of the polyps totally contracted (Fig. 3a). Exposure to rapamycin largely inhibited budding. In particular, budding of the polyps in 3 μM rapamycin was not observed in the strains other than J7apo. Figure 3b shows the changes in the number of polyps for each experimental condition two weeks after the start of the treatment, and transitions in the number of polyps for each condition during the treatment are shown in Supplementary Fig. S6. Polyps for each strain in the control condition gradually proliferated by budding (105: 15.4 ± 1.91; 105G: 12.4 ± 0.51; J7apo: 13.2 ± 0.58; J7: 10.4 ± 0.40, means ± SEM). The J7 strain formed buds slowly due to its relatively large polyps. Rapamycin treatment prevented the polyps of the 105 strain from budding (105 [1 μM]: 9.4 ± 0.24; 105 [3 μM]: 9.0 ± 0.45). The polyps of the J7apo could generate their buds under the rapamycin treatment, but the increase in the polyps was still suppressed (J7apo [1 μM]: 10.4 ± 0.51; J7apo [3 μM]: 9.8 ± 0.20). On the other hand, the polyps of the 105G strain were severely affected by rapamycin treatment. More than half of the polyps died under both 1 and 3 μM rapamycin (105G [1 μM]: 4.0 ± 0.84; 105G [3 μM]: 3.2 ± 0.86). The polyps of the J7 strain showed an intermediate-severe response to rapamycin treatment, falling between the 105 and 105G strains. Rapamycin at 1 μM impeded budding in the J7 strain, but most of the polyps survived during the treatment, similar to strain 105 (J7 [1 μM]: 8.2 ± 0.73). More than half of the polyps of the J7 strain died, similar to the 105G strain in 3 μM rapamycin (J7 [3 μM]: 4.4 ± 1.17). The increased mortality of the symbiotic polyps rather than non-symbiotic ones under the 3 μM rapamycin treatments indicates that rapamycin was more harmful to the symbiotic strains (105G, J7) than the non-symbiotic strains (105, J7apo). These results suggest that symbiosis with chlorococci alters their sensitivity to inhibition of translation and metabolism in host cells. In addition, we found higher mortality of 105G strain under 1 μM rapamycin treatments compared to that of J7. This indicates that the degree of sensitivity to rapamycin was greater in strain 105G than in J7, suggesting that strain J7 may have an adaptive mechanism to balance the cellular metabolism of hosts and chlorococci, which does not exist in 105G. Indeed, strain J7 did not show significant expression changes in the genes related to translation between the non-symbiotic and symbiotic strains, despite drastic changes in the host cell environment. The difference in the sensitivity to rapamycin treatment found between the acquired symbiotic strain 105G and the native symbiotic strain J7 might imply that the evolution of the ability to balance cellular metabolism between the host and the symbiont is one of the key requirements for adaptation to endosymbiosis with chlorococci.

**Figure 4:**
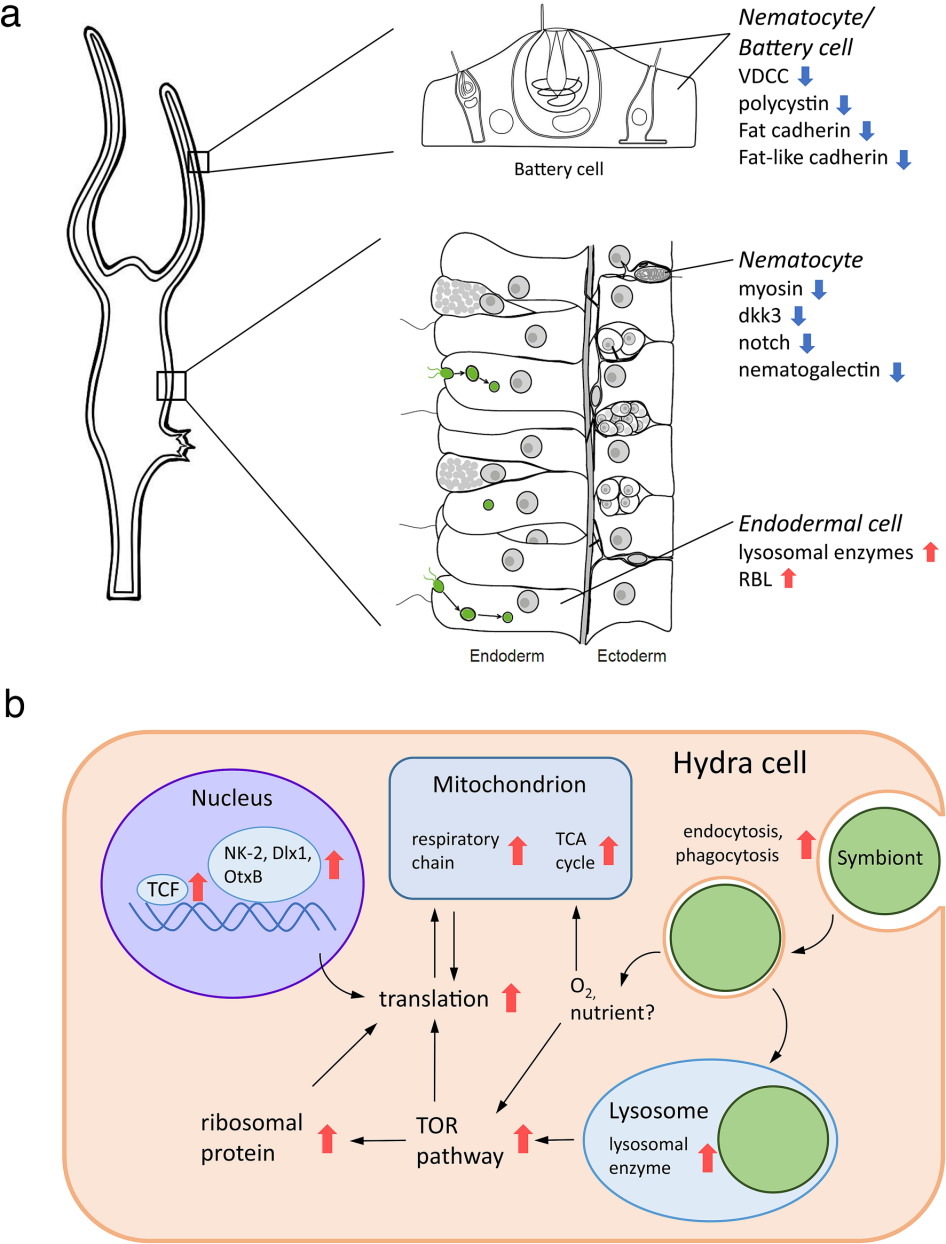
Summary of probable changes in the symbiotic hydra cells. The red arrows show the related genes that were upregulated, and the blue arrows show the related genes that were downregulated. (**a**) Common gene expression changes in symbiotic polyps in strains 105G and J7. (**b**) Cellular metabolism changes in strain 105G cells. The black arrows represent signalling and substance transmission.

Our study provided an overview of the symbiotic system in *H. vulgaris* from the changes in the gene expression patterns. In the symbiotic strains 105G and J7, similar gene expression patterns were found in genes related to uptake and maintenance of the symbionts, nematocyte differentiation, and development (Fig. 4a). These expression patterns indicate that these processes are likely to be essential mechanisms of brown hydra–chlorococcum symbiosis. On the other hand, the genes involved in translation and respiration tended to be upregulated only in the acquired symbiotic strain 105G (Fig. 4b). We also observed a difference between the non-symbiotic strain and the symbiotic strains regarding mortality caused by translation inhibition through rapamycin treatment. The difference in mortality between the acquired symbiotic strain and the native symbiotic strain suggested that the native symbiotic strain was more adapted to endosymbiosis with *Chlorococcum* than was the symbiotic strain acquired by horizontal transmission. Clarifying the unstable symbiotic mechanism may provide a better understanding of the evolution of symbiosis.

## Methods

### Materials

We employed three strains of *Hydra vulgaris* (formally described as *H. magnipapillata*): 105, 105G, J7, and J7apo. Strain J7 is a native symbiotic strain, and strain J7apo is an aposymbiotic strain originated from strain J7 whose symbiont was eliminated by keeping the polyp under dark condition^13^. Strain 105, J7, and J7apo were stored at the National Institute of Genetics (NIG; Mishima, Japan). Strain 105G is a symbiotic strain that originated from strain 105, which has established a symbiotic relationship with *Chlorococcum* sp. through horizontal transmission of the symbionts from a J7 polyp cultured in the same vessel^14^. The *rbcL* sequence of the symbionts in strain 105G was identical to that of the symbionts in strain J7^14^. All the strains were maintained in hydra culture solution﻿ (HCS; 1 mM NaCl, 1 mM CaCl_2_, 0.1 mM KCl, 0.1 mM MgSO_4_, 1 mM tris-(hydroxymethyl)-aminomethane; pH 7.4, adjusted with HCl) in glass vessels at 20 ﻿°C ﻿under 14 h∶10 h light/dark illumination cycles (14L/10D; 84 μmol/m^2^/s light intensity). ﻿Polyps were fed newly hatched Artemia nauplii two times a week. The day after feeding, the polyps were transferred into glass vessels with fresh HCS.

### RNA extraction and sequencing

Hydra polyps, after being starved for three days, were used for RNA-seq analysis to remove the effect of nutrient factors on the gene expression from the prey. Total RNA was extracted from intact polyps (30 polyps of strain 105 and 50 polyps of strain 105G) using the acid guanidinium thiocyanate–phenol–chloroform (AGPC) method^64^. Three biological replicates were prepared for both strains. Total RNA was treated with DNase I (Roche, Mannheim, Germany) to remove genomic DNA. The total RNA samples were sent to Novogene Co., Ltd. (Beijing, China) for cDNA library construction and sequencing of 150 bp paired-end reads on Illumina HiSeq 4000.

### Mapping and differential gene expression analysis

Quality trimming of reads was performed using cutadapt^65^. Low-quality ends (QV < 30) and adapter sequences were trimmed, and short reads (< 20 bp) were discarded, for quality control. The trimmed reads were aligned to the *Hydra* genome reference (GCA_000004095.1)^66^ using TopHat2 (version 2.1.1)^67^. We estimated gene expression levels based on fragments per kilobase of exon per million mapped reads (FPKM) using the Cufflinks (version 2.2.1) pipeline^68^. All transcriptome samples were merged into contigs using Cuffmerge, and then Cuffdiff was used to normalize the read counts of each sample and to analyse differential gene expression between strains 105 and 105G. The contigs were considered differentially expressed if they showed a false discovery rate (FDR) < 0.05. To compare strain 105G with native symbiotic strain J7, we used RNA-seq data of strain J7 and J7apo (symbionts eliminated strain J7) analysed by Ishikawa et al.^13^ We obtained raw sequence data of J7 and J7apo strains (PRJDB4331) and carried out quality trimming for these data using the same criteria as for strain 105 and 105G described above. The trimmed reads were mapped to the *Hydra* genome reference in the same way as those of 105 and 105G. We also performed differential gene expression analyses of each strain with the -b/-frag-bias-correct Cuffdiff command-line option for comparison of changes in expression patterns between 105G/105 and J7/J7apo to normalize read counts.

### Gene ontology (GO) analysis

The contigs were subjected to a similarity search against the UniProtKB Swiss-Prot and *H. vulgaris* proteins in the UniProtKB TrEMBL using BLASTX with an e-value cutoff of 1e-5. GO enrichment analysis was performed using the DAVID Functional Annotation tool^69^. UniProt accessions annotated to the contigs were entered into DAVID as queries. GO terms were considered to be significantly enriched if they showed adjusted P (Benjamini) < 0.05 by Fisher’s exact test. The R package pathview^70^ was used to conduct a pathway analysis for several KEGG pathways^71,72^ (https://www.genome.jp/kegg/pathway.html), including the differentially expressed genes (DEGs).

### Rapamycin treatment

Rapamycin (Funakoshi, Tokyo) was dissolved in dimethyl sulfoxide (DMSO) to 1 mM or 3 mM as a stock solution. Next, each rapamycin stock solution was dissolved in 6 mL HCS to make 1 μM or 3 μM solutions. For the control condition, 0.1% DMSO in HCS was used. Ten budless polyps for each condition were used at the start of the experiments and cultured in plastic containers filled with rapamycin solutions at 20 °C under 14 h∶10 h light/dark illumination cycles (14L/10D; 15 μmol/m^2^/s light intensity) for two weeks. The numbers of live polyps under each condition were counted per day. The polyps were fed *Artemia* nauplii on days 0, 4, 7, and 11. The polyps were observed with a stereomicroscope (MZ75, Leica, Wetzlar, Germany) and photographed with a digital camera (Digital Sight DS-L1, Nikon, Tokyo). The number of polyps in each condition or strain was tested by Tukey’s test.

## Supplementary Information

Supplementary Tables.

Supplementary Figures.

## Data Availability

The datasets generated during the current study are available in the DDBJ Sequence Read Archive (https://www.ddbj.nig.ac.jp/dra/index.html) under accession number DRA010715.
