# Supplementary material for: Immature symbiotic system between horizontally transmitted green algae and brown hydra
Source: Sci Rep. 2021 Feb 3;11:2921. doi: 10.1038/s41598-021-82489-6 (PMC7859245; doi:10.1038/s41598-021-82489-6)
Supplement: Supplementary file 2 — Supplementary Figures. [file 41598_2021_82489_MOESM2_ESM.pdf]

Immature symbiotic system between horizontally transmitted green algae and brown hydra

Ryo Miyokawa[1], Hiroyuki J. Kanaya[2], Taichi Q. Itoh[3], Yoshitaka Kobayakawa[3] and Junko Kusumi[4*]

[1]Graduate School of Integrated Science for Global Society, Kyushu University, 744 Moto-oka, Nishi-ku, Fukuoka 819-0395, Japan
[2]School of Science, Kyushu University, 744 Moto-oka, Nishi-ku, Fukuoka 819-0395, Japan
[3]Faculty of Arts and Science, Kyushu University, 744 Moto-oka, Nishi-ku, Fukuoka 819-0395, Japan
[4]Faculty of Social and Cultural Studies, Kyushu University, 744 Moto-oka, Nishi-ku, Fukuoka 819-0395, Japan

[*]Corresponding author
TEL/FAX; +81-92-802-5649
E-mail: jkusumi@scs.kyushu-u.ac.jp

References

1. Kanehisa, M. & Goto S. KEGG: kyoto encyclopedia of genes and genomes. *Nucleic Acids Res*. 28, 27-30 (2000).
2. Kanehisa M. Toward understanding the origin and evolution of cellular organisms. *Protein Sci.* 28, 1947-1951(2019).

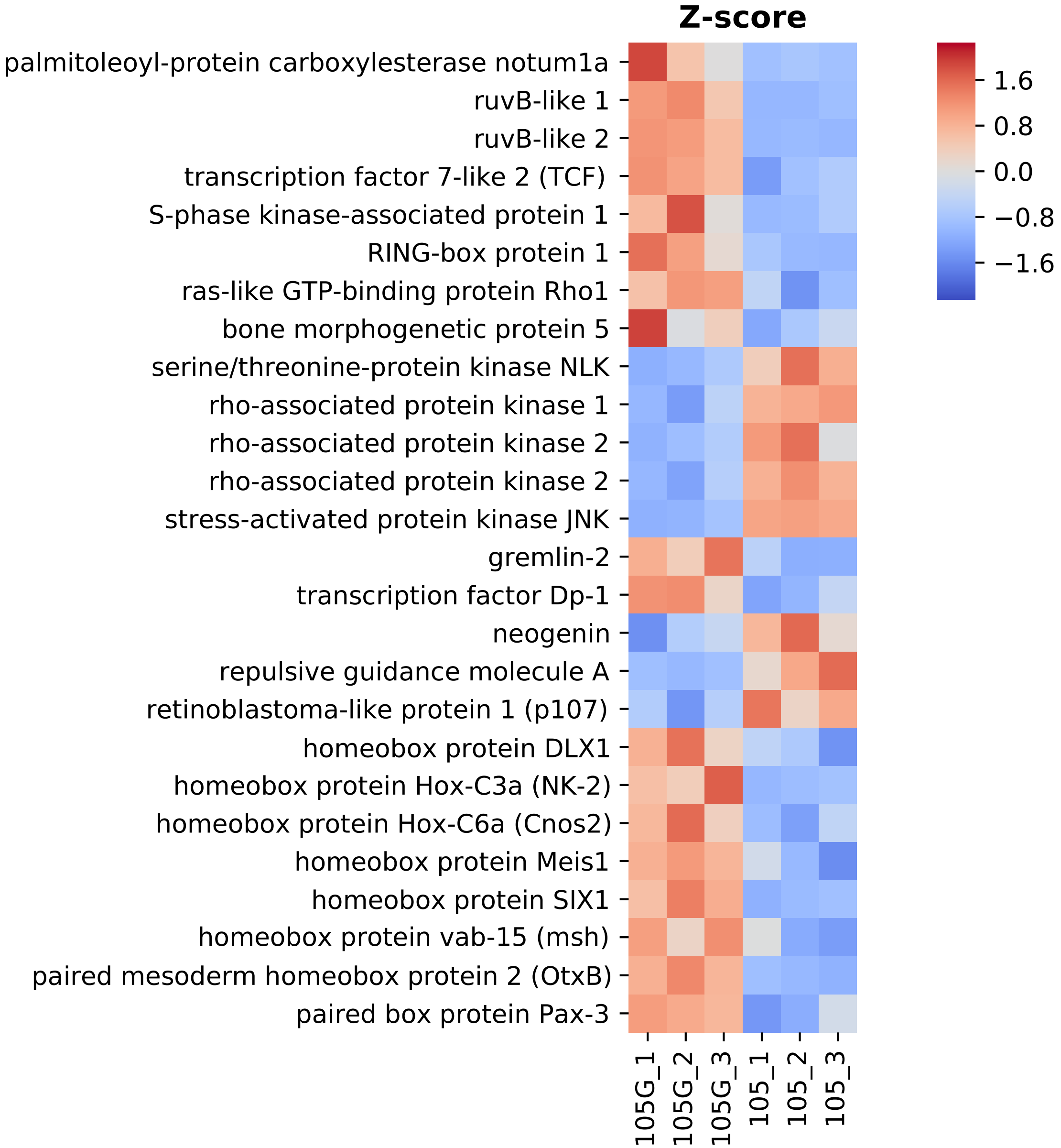

Fig. S1. Heat map showing gene expression patterns involved in the Wnt pathway and homeobox genes in strains 105G and 105.
The Z-scores are calculated from the FPKMs of each gene. The displayed expressions are selected notable genes' (FDR < 0.05).

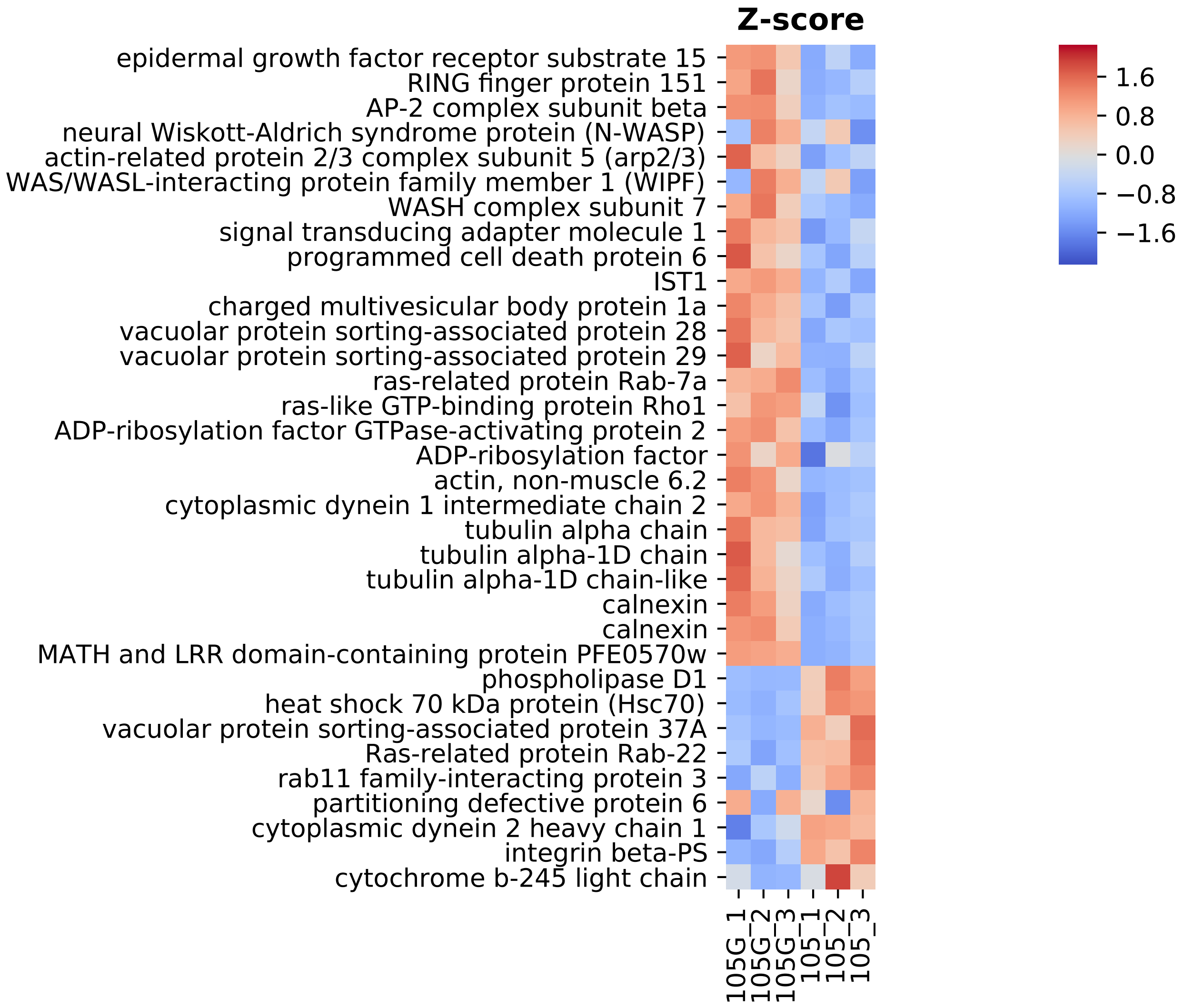

Fig. S2. Heat map showing gene expression patterns involved in endocytosis and phagocytosis pathways in strains 105G and 105.

The Z-scores are calculated from the FPKMs of each gene. The displayed expressions are selected notable genes' (FDR < 0.05).

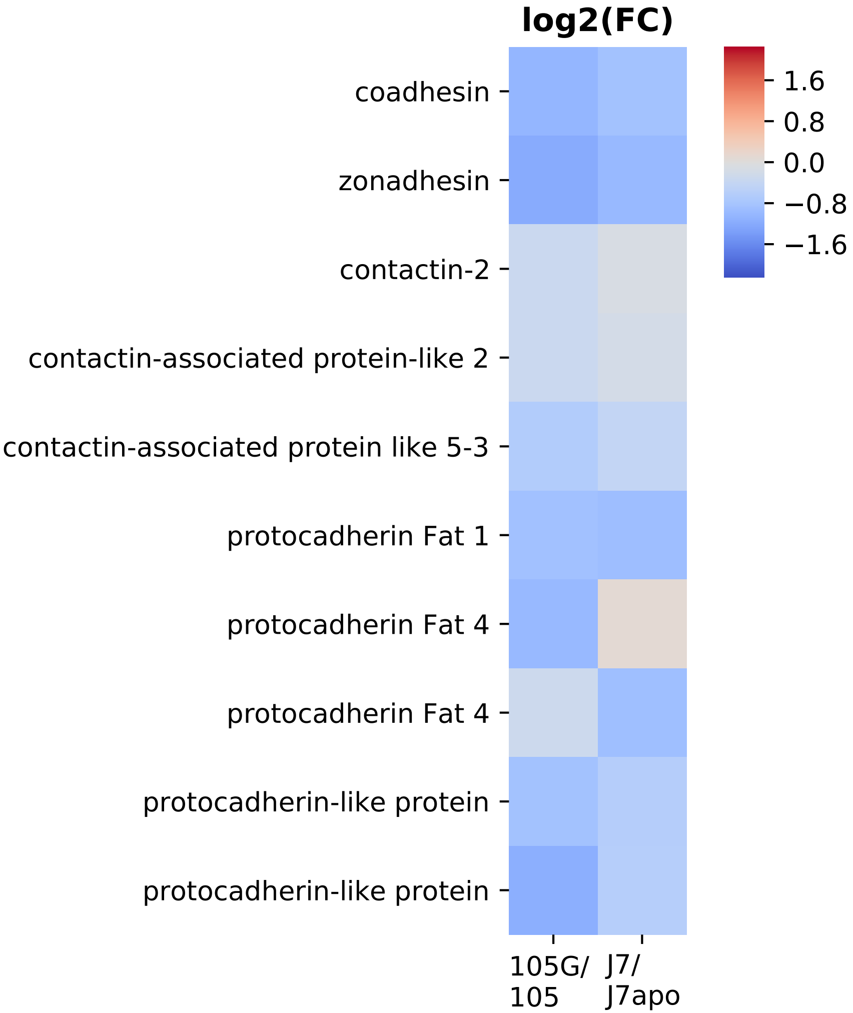

Fig. S3. Heat map showing fold changes of genes involved in cell adhesion in 105G/105 and J7/J7apo pairs.
The fold changes are calculated from the FPKMs of each strain pair. The displayed expressions are selected notable genes' (FDR < 0.05).

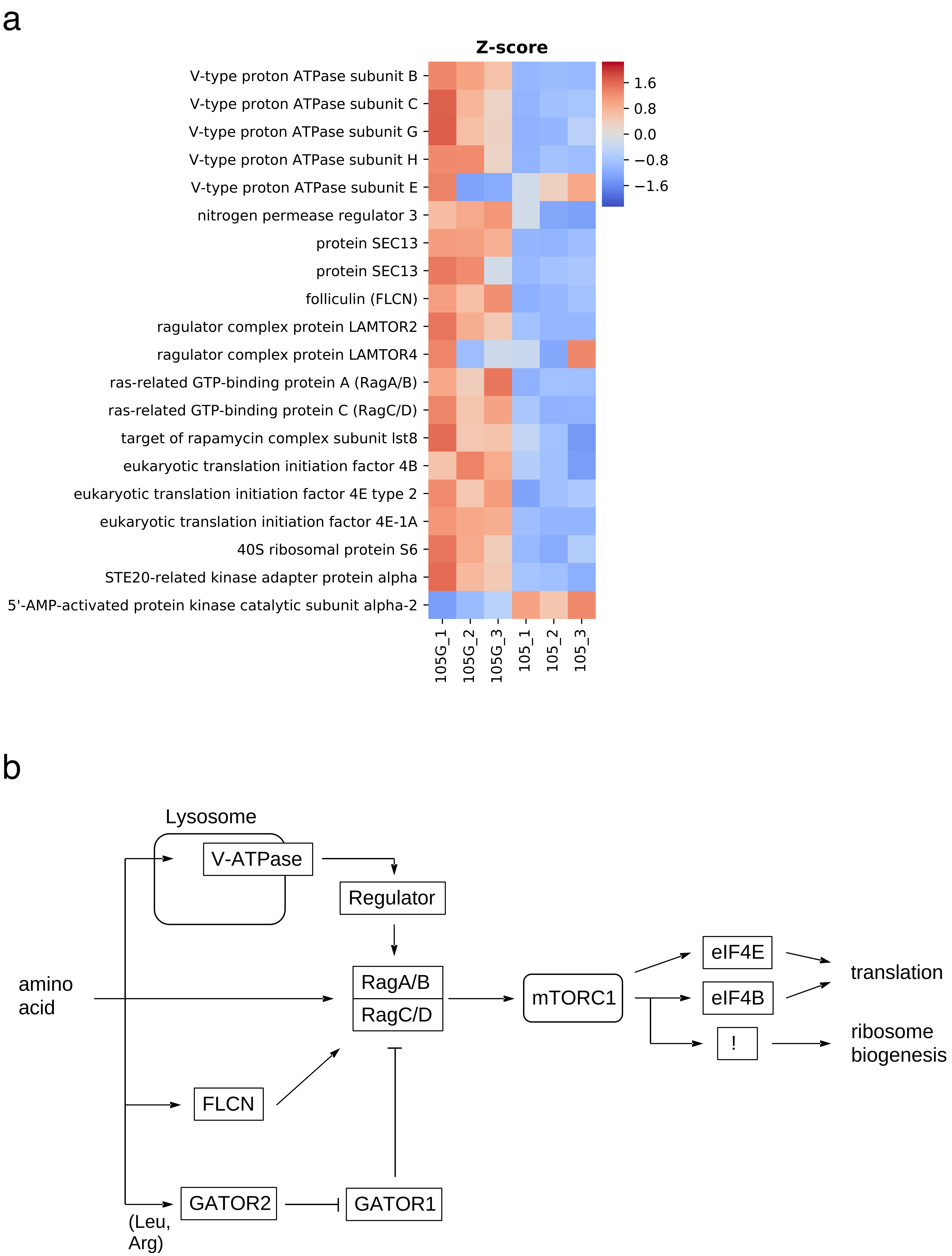

Fig. S4. Gene expression changes involved in the TOR pathway.
(a) Heat map showing the expression patterns of the genes in strains 105G and 105. The Z-scores are calculated from the FPKMs of each gene. The displayed expressions are selected notable genes' (FDR < 0.05). (b) Overview of the TOR pathway based on DEGs in strain 105G.[1,2] Genes enclosed in squares represent upregulated genes in strain 105G.

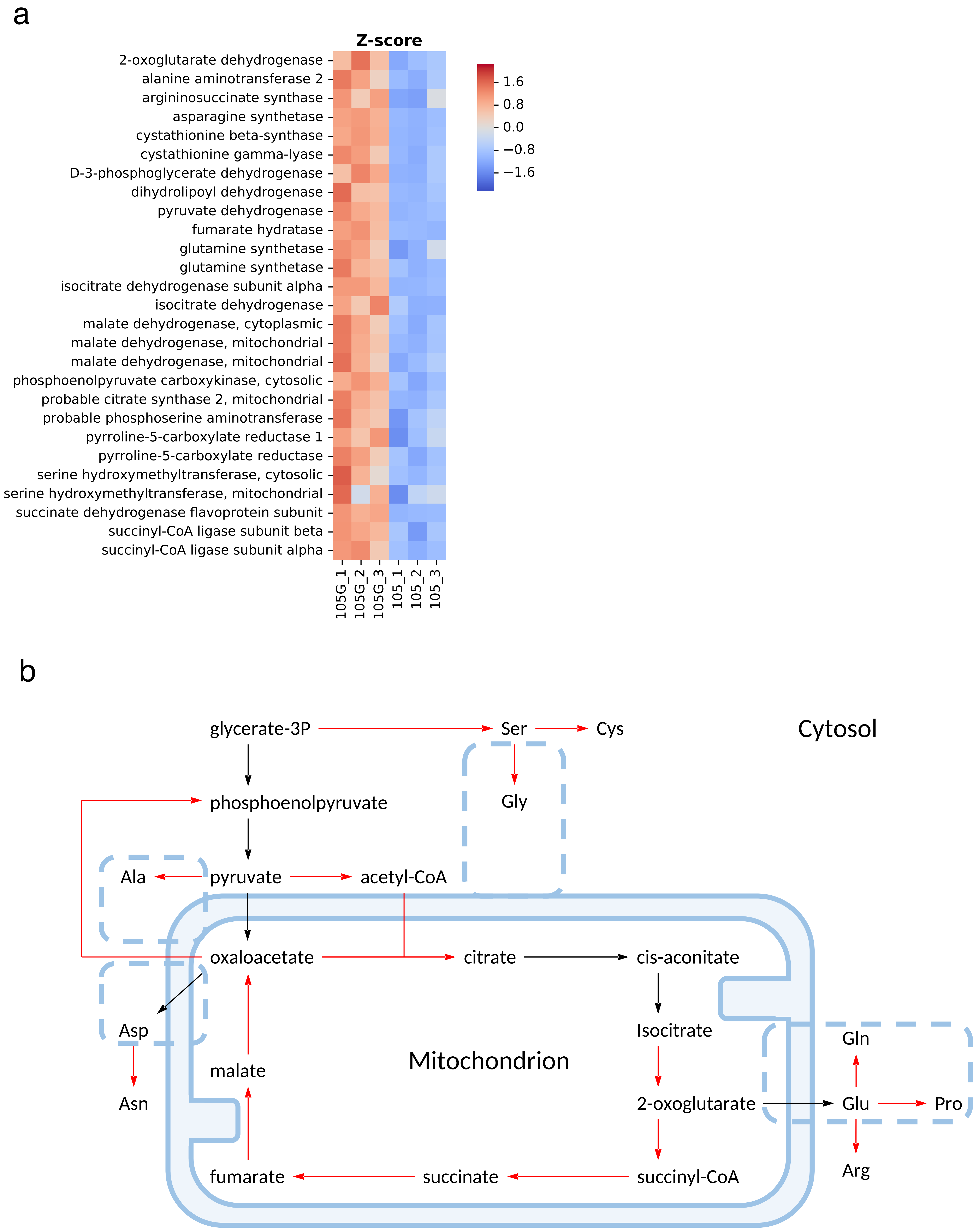

Fig. S5. Expression changes in genes encoding enzymes that synthesize amino acids and are involved in the TCA cycle.
(a) Heat map showing fold changes of the genes in strains 105G and 105. The Z-scores are calculated from the FPKMs of each gene (FDR < 0.05).
(b) Overview of the TCA cycle and the pathway of amino acid synthesis based on DEGs in strain 105G.[1,2] Red arrows represent enzymes coded in upregulated genes in strain 105G, and black arrows represent enzymes coded in genes not showing significant differences in strain 105G.
Rounded rectangles with dashed lines show that amino acids inside the rectangles are synthesized in both mitochondria and cytosol.

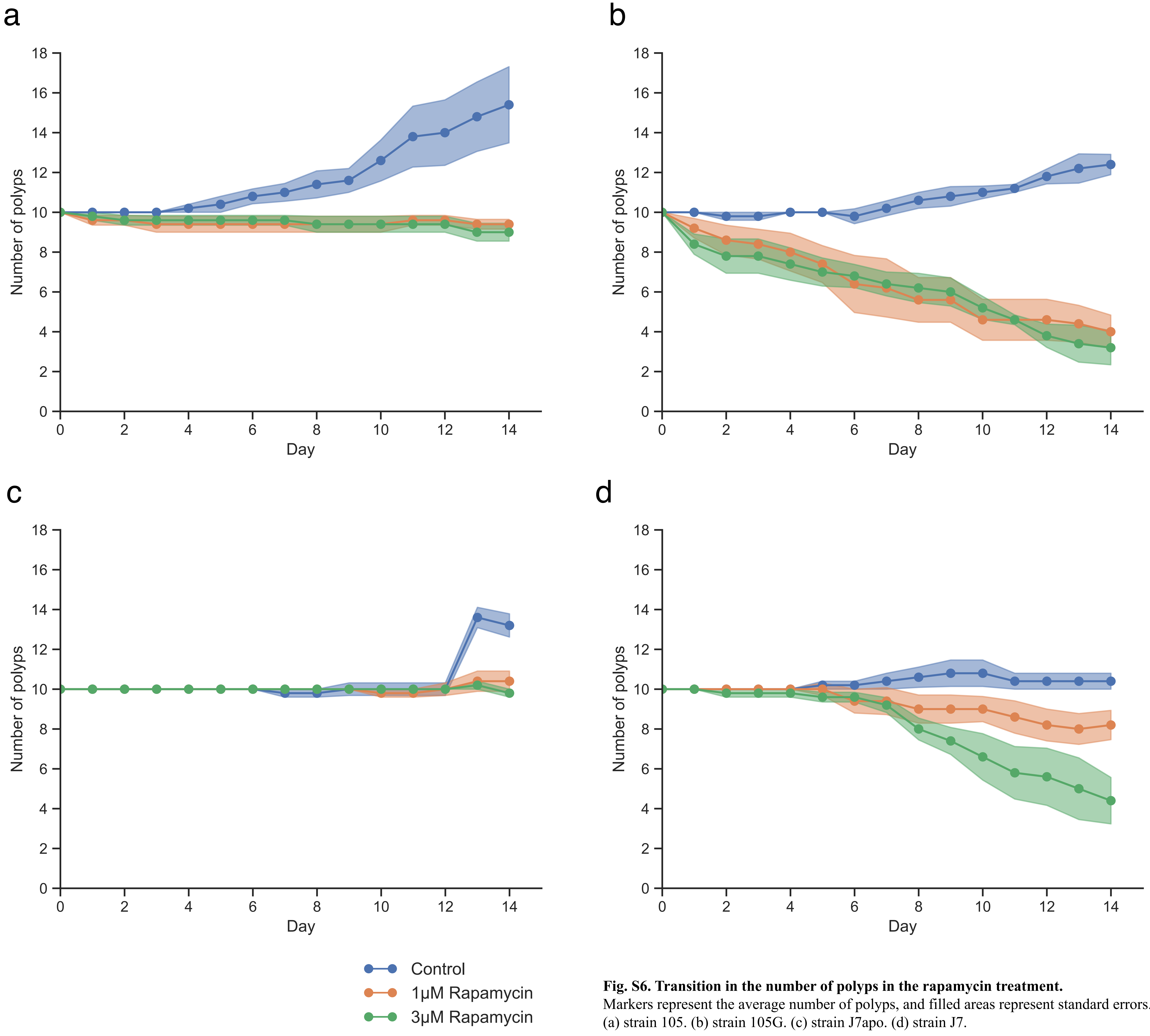

Fig. S6. Transition in the number of polyps in the rapamycin treatment.
Markers represent the average number of polyps, and filled areas represent standard errors. (a) strain 105. (b) strain 105G. (c) strain J7apo. (d) strain J7.
